# Age-Related Volumetric Changes of the Posterior Cranial Fossa and its Structures in Stroke Patients

**DOI:** 10.1007/s12311-026-02082-3

**Published:** 2026-09-11

**Authors:** Artem Rafaelian, Daniel Cantré, Daniel Dubinski, Enayatullah Baki, Tim-Mathis Beutel, Nazife Dinc, Judith Dremel, Nima Etminan, Thomas M. Freiman, Kiarash Ferdowssian, Florian Gessler, Erdem Güresir, Katharina A.M. Hackenberg, Bernhard Hemmer, Silvia Hernández-Durán, Zin Yu Hlaing, Dragan Jankovic, Andreas Kramer, Sandro M. Krieg, Wendy H. Nabo, Paul Naser, Anne Neumeister, Florian Ringel, Veit Rohde, Daria Samoylenko, Daniela Marin-Sanabria, Jan Hendrik Schäfer, Patrick Schuss, Michael Schwake, Christian Senft, Santhosh G. Thavarajasingam, Moritz Thiel, Peter Vajkoczy, Martin Vychopen, Johannes Wach, Johannes Walter, Silke Wunderlich, Matthias Wittstock, Sae-Yeon Won

**Affiliations:** 1 Department of Neurosurgery, University Medicine Center Rostock, Schillingallee 35, 18057 Rostock, Germany; 2 Department of Neurosurgery, Charité –Universitätsmedizin Berlin, corporate member of Freie Universität Berlin, Humboldt-Universität zu Berlin and Berlin Institute of Health, Berlin, Germany; 3 Department of Neuroradiology, School of Medicine and Health, Klinikum Rechts der Isar, Technical University of Munich, Munich , Germany; 4 Institute and Policlinic for Radiology, Paediatric Radiology and Neuroradiology, University Medical Center Rostock, Rostock, Germany; 5 Department of Neurosurgery, University Hospital Jena, Jena, Germany; 6 Department of Neurosurgery, University Hospital Mannheim, Mannheim, Germany; 7 Department of Neurology, School of Medicine and Health, Klinikum Rechts der Isar, Technical University of Munich, Munich, Germany; 8 Department of Neurosurgery, University Hospital Leipzig, Leipzig, Germany; 9 Department of Neurosurgery, LMU University Hospital, LMU Medizin, Ludwig-Maximilians-Universität München, Munich, Germany; 10 Department of Neurosurgery, Heidelberg University Hospital, Heidelberg, Germany; 11 Medical Faculty of Heidelberg University, Heidelberg, Germany; 12 Department of Neurosurgery, University Hospital Göttingen, Göttingen, Germany; 13 Department of Interventional and Diagnostic Neuroradiology, University Hospital Göttingen, Göttingen, Germany; 14 Department of Neurology, Goethe University Frankfurt, University Hospital, Frankfurt am Main, Germany; 15 Department of Neurosurgery, BG Klinikum Unfallkrankenhaus, Berlin, Germany; 16 Department of Neurosurgery, University Hospital Münster, Münster, Germany; 17 Department of Neurology, University Medical Center Rostock, Rostock, Germany

**Keywords:** Posterior fossa, cerebellum, Brainstem, Aging, MRI volumetry

## Abstract

The posterior cranial fossa (PCF) contains critical neural structures, including the cerebellum and brainstem, whose volumetric relationships play an essential role in normal neurological function. While cranial bone growth is completed in early adulthood, brain structures may undergo age-related changes, particularly prominent in patients with stroke, potentially altering craniocerebral relationships within the PCF. Data on volumetric changes of the PCF and its contents in the adult population are limited. The aim of the study was to evaluate age- and sex-related volumetric changes of the PCF and its main neural structures in adults using MRI- and CT-based volumetric analysis.This retrospective, multicenter study included 1,280 adult subjects who underwent neuroimaging for suspected or confirmed cerebellar stroke at 11 university hospitals in Germany. In most centers, segmentation and volumetric analysis were performed using Brainlab software (Brainlab AG, Munich, Germany). In selected centers, volumetric calculations were performed using the standard method according to local protocol.PCF, cerebellar, and brainstem volumes demonstrated significant age-related changes. Cerebellar volume showed a progressive decline with increasing age from 136 cm3 (IQR 123.6–147.2) to 115.2 cm3 (IQR 107.4–131.1) (p < .001), whereas posterior fossa cerebrospinal fluid volume (PCF-CSFv) increased, indicating age-related expansion of infratentorial cerebrospinal fluid spaces. Across most age groups, males exhibited larger PCF, cerebellar, and brainstem volumes than females. In the overall cohort, these differences remained statistically significant after Holm–Bonferroni correction (all p-corrected < 0.001), whereas PCF-CSFv did not differ significantly between females and males (p-corrected = 0.438).Aging is associated with a progressive reduction of infratentorial neural tissue volumes, particularly affecting the cerebellum, accompanied by a relative expansion of PCF-CSFv. Pronounced sex-related volumetric differences persist throughout adulthood, with males demonstrating larger infratentorial structures than females. These findings provide robust normative data that may aid in the interpretation of PCF pathology in clinical and research settings.

## Introduction

The infratentorial compartment of the cranial cavity, defined as the posterior cranial fossa (PCF), lodges critical regions of the central nervous system including the cerebellum and brainstem [1]. These structures are situated below the tentorium cerebelli and are bounded by the occipital, temporal, and sphenoid bones [2]. The brainstem, which consists of the midbrain, pons, and medulla, occupies the anterior portion of the PCF and is crucial for maintaining life-sustaining functions. The fourth ventricle, a cerebrospinal fluid (CSF)-filled cavity, lies between the dorsal surfaces of the brainstem and the cerebellum, forming part of the ventricular system. Changes in the volume of cerebrospinal fluid spaces have been found in healthy elderly people and can be considered compensatory spaces [3].

Volumetric relationships between the bony boundaries of the PCF and its neural contents are essential for normal neurological functioning, as they influence the position and morphology of vital structures within this confined space. Within this framework, the concept of infratentorial “reserve space” becomes particularly relevant. In the posterior cranial fossa, acute space-occupying pathologies—such as cerebellar infarction, hemorrhage, edema, or postoperative swelling—initially lead to displacement of cerebrospinal fluid before direct compression of neural tissue occurs [4]. However, data on normative age-related volumetric changes of the PCF and its contents in adults remain limited and somewhat inconsistent in the literature [5,6] It is well established that cranial bone growth is completed in early adulthood, yet neural structures including the cerebellum undergo age-dependent atrophic changes [7]. Such changes can alter craniocerebellar relationships without significant changes in the osseous boundaries of the fossa, especially in later decades of life.

Several studies have demonstrated that limited infratentorial volume and unfavorable volumetric ratios are associated with an increased risk of malignant cerebellar swelling and clinical deterioration [8]. Likewise, volumetric ratios between lesion size and available infratentorial space have been shown to correlate with the need for surgical decompression and adverse outcome [9]. These findings underline that not only infarct size itself but also the anatomical capacity of the posterior cranial fossa critically determines the clinical course.

Therefore, studying age-related volumetric changes of the PCF and its neural structures using advanced volumetric methods is a timely and relevant goal in neuroanatomy and neuroradiology. Against this background, the present study was designed to systematically characterize age- and sex-related volumetric differences of the PCF and its contents in a large cohort of adult patients undergoing neuroimaging for cerebellar stroke. By providing reference infratentorial volumetric data derived from a clinically relevant vascular population, we aim to contribute to a more individualized framework for interpreting critical volumes and reserve capacity in PCF pathology, particularly in the context of acute cerebrovascular disease.

## Materials and Methods

This multicenter retrospective study included 1,280 adult patients aged 18 to 103 years who underwent brain imaging for suspected or confirmed cerebellar stroke at 11 university hospitals in Germany between January 2010 and January 2024. Imaging was performed using either magnetic resonance imaging (MRI) or computed tomography (CT), according to local clinical protocols [10]. The study was conducted in accordance with the Declaration of Helsinki [11]. MRI examinations were based on high-resolution T1-weighted sequences, providing optimal anatomical contrast for delineation of infratentorial structures. In patients undergoing CT, thin-slice axial reconstructions were used for volumetric assessment.

### Volumetric Analysis

**Fig. 1:**
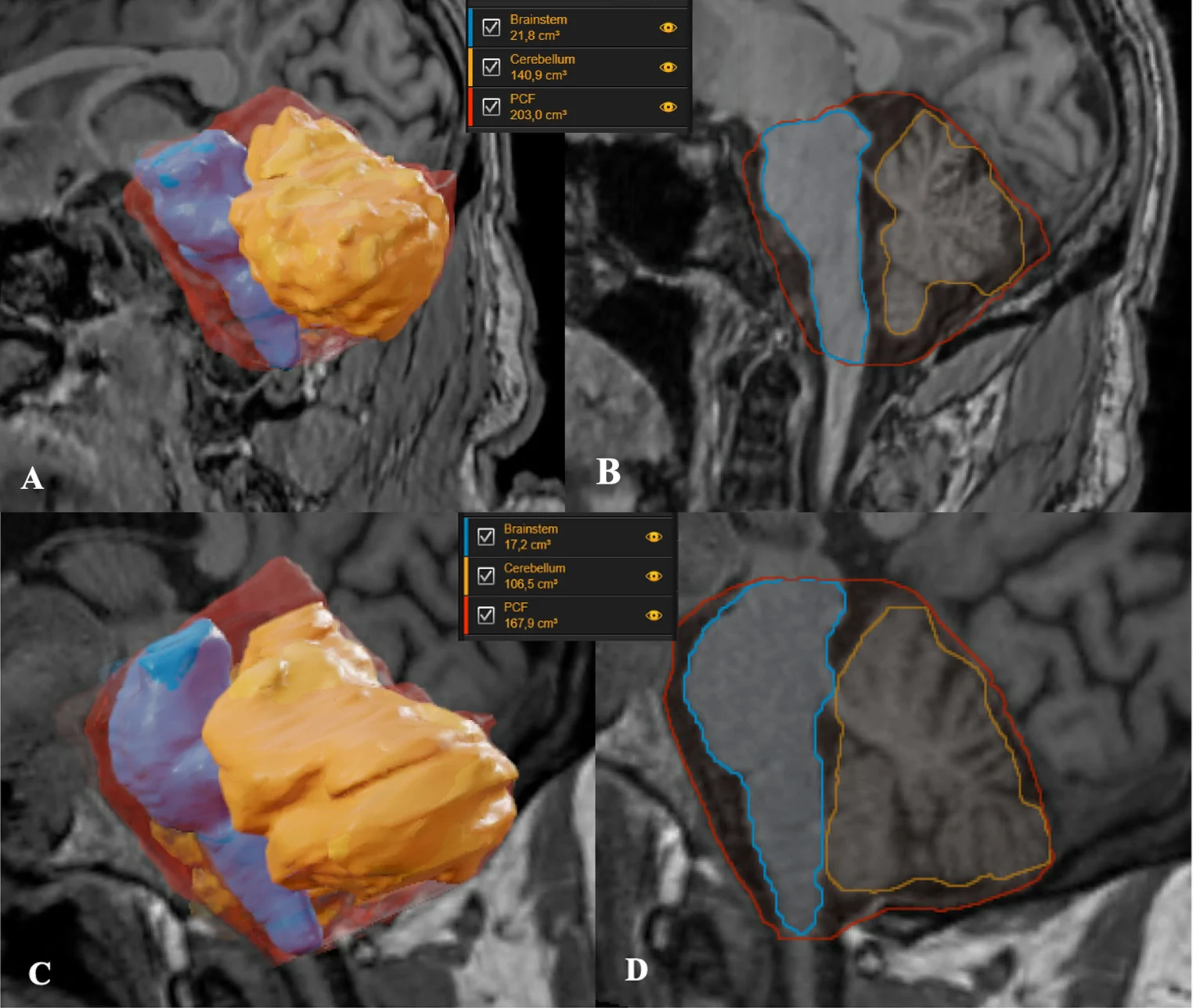
Representative examples of semi-automated volumetric segmentation of the posterior cranial fossa (PCF), cerebellum, and brainstem using Brainlab Segmentation software. (**A**–**B**) Segmentation in a 48-year-old male subject. (**A**) Three-dimensional reconstruction illustrating the segmented posterior cranial fossa (red), cerebellum (yellow), and brainstem (blue). (**B**) Corresponding sagittal T1-weighted MR image demonstrating anatomical boundaries and segmentation contours. (**C**–**D**) Segmentation in an 89-year-old female subject. (**C**) Three-dimensional reconstruction showing age-related reduction of infratentorial parenchymal structures and relative enlargement of the posterior fossa cerebrospinal fluid space. (**D**) Corresponding sagittal T1-weighted MR image with segmentation contours of the posterior cranial fossa (red), cerebellum (yellow), and brainstem (blue)

All volumetric measurements were performed in the subjects’ native anatomical space and were based on direct segmentation of predefined anatomical structures; therefore, spatial normalization to a standard template was not applied [12]. In most participating centers, volumetric segmentation was performed using Brainlab Elements SmartBrush version 5.0.0.165 and Object Management version 5.0.0.165 (Brainlab AG, Munich, Germany). SmartBrush was used for semi-automated, interactive delineation of anatomical structures and generation of three-dimensional volumetric objects. (Fig. 1). In two centers, volumetric calculations were performed using the standard method according to institutional protocol [13]. All volumetric measurements were expressed in cubic centimeters (cm³). The following parameters were assessed:

- *PCF volume*, defined as the infratentorial compartment bounded anteriorly by the clivus,laterally by the petrous temporal bones, posteriorly by the occipital bone, and superiorlyby the tentorium cerebelli. The superior boundary was operationally defined by a virtualplane extending from the tentorial apex toward the clivus, approximating the level of theposterior commissure [14];
- *Cerebellar volume*, including both cerebellar hemispheres and the vermis;
- *Brainstem volume*, comprising the midbrain, pons, and medulla oblongata;
- *Infarct volume*, segmented separately based on diffusion-weighted imaging

In addition to absolute volumetric measurements, the posterior fossa cerebrospinal fluid volume (PCF-CSFv) was calculated. This parameter was defined as the difference between the total posterior cranial fossa volume and the combined volumes of the cerebellum and brainstem. PCF-CSFv represents the cerebrospinal fluid–containing spaces within the posterior cranial fossa, including the cisternal compartments and the fourth ventricle, and was used as a quantitative surrogate of infratentorial reserve space.

### Statistical Analysis

Descriptive statistical analysis was performed using Microsoft Excel (Microsoft Corporation, Redmond, WA, USA). Continuous variables were summarized as median ± interquartile range. Group comparisons across age categories were performed using one-way analysis of variance (ANOVA) with Tukey’s post hoc test for multiple comparisons, implemented in GraphPad Prism 10 (GraphPad Software, San Diego, CA, USA). Sex-related differences within individual age groups were assessed using the Mann-Whitney U test. To control the family-wise error rate, p-values for the age-stratified sex comparisons presented in (Fig. 3) were adjusted using the Holm–Bonferroni procedure separately for each volumetric parameter across the six age groups. For the overall sex comparisons presented in (Table 2), Holm–Bonferroni correction was applied across the four volumetric parameters. Analyses were performed using available cases. A corrected p-value (p-corrected) < 0.05 was considered statistically significant.

## Results

### Study Cohort Characteristics

**Fig. 2:**
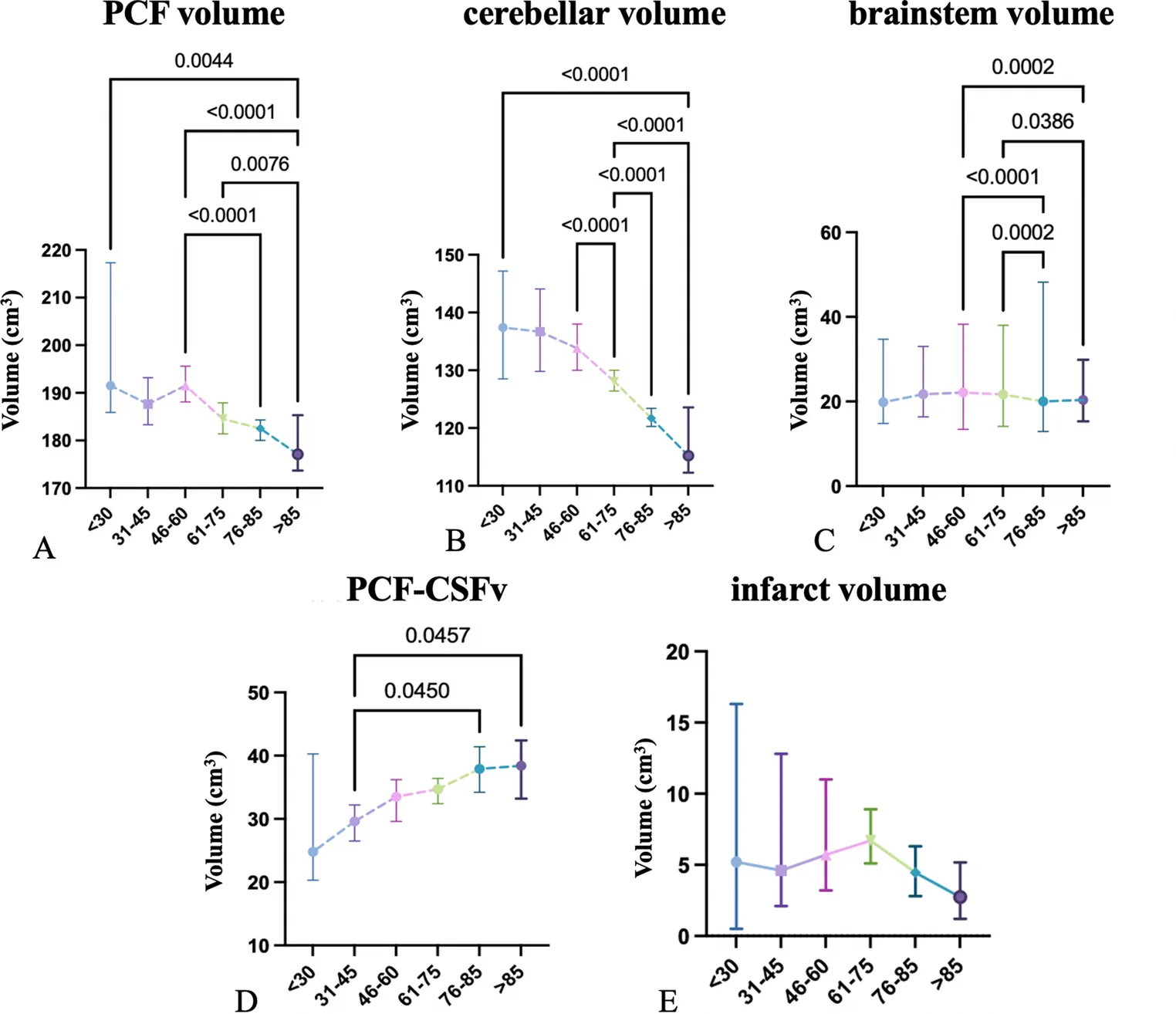
Age-related differences in infratentorial volumes across predefined age groups: (**A**) Posterior cranial fossa (PCF) volume, (**B**) cerebellar volume, (**C**) brainstem volume, and (**D**) PCF fluid volume (PCF-CSFv) and (**E**) infarct volume. Data are presented as median with interquartile range (25th-75th percentile). Between-group comparisons were performed using one-way ANOVA with post hoc multiple comparisons; brackets indicate statistically significant differences with corresponding p-values

A total of 1280 adult subjects aged were included in the analysis. The cohort was stratified into six age groups: 18–30 years (*n* = 22, 1.7%), 31–45 years (*n* = 104, 8.1%), 46–60 years (*n* = 245, 19.1%), 61–75 years (*n* = 459, 35.9%), 76–85 years (*n* = 366, 28.6%), and > 85 years (*n* = 84, 6.6%). Both female and male subjects were represented across all age groups. A complete overview of volumetric characteristics of the study is presented in (Table 1). Total PCF volume demonstrated a progressive decline with increasing age (Fig. 2). Median PCF volume decreased from 191.7 cm³ (IQR 186.1-222.9) in the 18–30 years group to 177.1 cm³ (IQR 164.9-192.4) in subjects older than 85 years. One-way analysis of variance revealed a significant overall effect of age on PCF volume, with significant differences observed between younger and older age groups. Across all age categories, males exhibited significantly larger PCF volumes compared to females, with statistically significant sex-related differences observed in most age groups.

**Table 1:** Baseline volumetric characteristics of the study cohort across age group

		Count, *n* (%)	Age, years median (IQR)	PCF volume, cm^3^ median, IQR	Cerebellar volume, cm^3^ median, IQR	Brainstem volume, cm^3^ median, IQR	PCF-CSFv, cm^3^ median, IQR	Infarct volume, cm^3^ median, IQR
Age group		*n* = 1280	70 (58–79)					
1 (18–30 years)		22 (1.7)	26 (24–28)	191.7 (186.1–222.9)	137.4 (128.2–147.2)	20.1 (19–23.7)	28.9 (22.7–41.2)	5.2 (0.5–16.3)
Female		12 (54.5)	25 (24–26)	189.5 (185.8–221.4)	130.1 (125.4–136.5)	19.8 (18.5–20.6)	36.3 (34.2–41.7)	4.03 (0.18–7.28)
Male		10 (45.5)	28 (26–30)	197.4 (190.7 -222.5)	147.3 (141.9–174.1)	23.6 (19.4–28.3)	26.6 (22.7–39.6)	5.2 (2.57–16.5)
p-value			0.091	0.456	0.042	0.123	0.601	0.421
2 (31–45 years)		104 (8.1)	41 (36–43)	187.7 (178.1–205.7)	136.7 (123.6–153.7)	21.7 (18.9–25.1)	29.6 (17.5–42.8)	4.6 (0.5–30)
Female		39 (37.5)	41 (36–44)	179.5 (170.3–194)	127.6 (115.8–143)	20.4 (18.4–21.6)	32.3 (17.9–44.9)	3 (1.25–30.7)
Male		65 (62.5)	40 (36–43)	191.4 (180.5–209.9)	144.1 (128.1–156.9)	23.9 (19.7–26.9)	29.5 (13.1–39.4)	4.9 (0.2–30)
p-value			0.426	0.004	< 0.001	< 0.001	0.449	0.504
3 (46–60 years)		245 (19.1)	54 (51–57)	190.2 (177.1–207.7)	133.8 (122.4–149.8)	22.1 (20–25.9)	31.5 (17.4–42.4)	5.7 (0.4–28.6)
Female		60 (24.5)	54 (50–57)	182.5 (167.7–199.2)	128.7 (118.5–141.1)	21.4 (18.3–23.6)	32.7 (18.3–41.8)	4.9 (0.4–30.1)
Male		185 (75.5)	54 (51–57)	191.2 (180.3–209.7)	144 (128–156.7)	23.7 (19.6–26.9)	29.5 (17.3–42.8)	6.0 (0.3–27.3)
p-value			0.11	0.001	0.004	0.005	0.932	0.638
4 (61–75 years)		459 (35.9)	69 (65–72)	184.4 (170.9–201.6)	128.1 (116.3–140.5)	21.8 (18.8–25)	34.7 (22–45.5)	6.1 (0.6–22.5)
Female		162 (35.3)	70 (66–72)	177 (166.4–189.7)	123.1 (112.1–132.9)	20.1 (17.9–23.4)	33 (21.4–45.2)	6.0 (0.1–21.6)
Male		297 (64.7)	69 (64–72)	188.5 (175.1–206.1)	131.6 (119.3–145.2)	22.2 (19.8–25.5)	35.6 (22.4–45.6)	6.5 (1.0–25.1)
p-value			0.128	< 0.001	< 0.001	< 0.001	0.455	0.146
5 (76–85 years)		366 (28.6)	80 (78–82)	182.5 (168.2–196.2)	121.7 (110–135.2)	20 (18–23.3)	37.9( 21.8–49.9)	4.5 (0.2–20.6)
Female		164 (44.8)	80 (78–82)	174 (161.8–188)	116.9 (106.9–130.4)	19.2 (17.4–21.2)	34.5 (19.8–46.2)	2.4 (0.2–19.2)
Male		202 (55.2)	80 (78–82)	187 (174.7–202.1)	124.9 (112.8–137.7)	21.2 (18.6–24.8)	40.9 (24.4–52.7)	5.6 (0.4–20.2)
p-value			0.887	< 0.001	< 0.001	< 0.001	0.051	0.212
6 (> 85 years)		84 (6.6)	88 (87–89)	177.1 (164.9–192.4)	115.2 (107.4–131.1)	20.4 (18.2–23)	38.4 (22.6–48)	3.8 (0.3–13.2)
Female		52 (61.9)	88 (87–90)	173.7 (162.2–184.3)	114.1 (105.6–126.0)	20.1 (17.6–22.2)	36.6 (21.6–46.9)	3.5 (0.2–15.3)
Male		32 (38.1)	87 (87–89)	186.1 (174.1–193.5)	123.3 (110.2–134.8)	20.9 (18.7–24.2)	41.5 (29.9–51.7)	4.8 (1.8–21.1)
p-value			0.122	0.005	0.01	0.09	0.398	0.189

Unadjusted p-values represent comparisons between male and female subjects within each age group (Mann-Whitney U test).

Abbreviations: *PCF-CSFv * posterior fossa cerebrospinal fluid volume, *PCF* posterior cranial fossa *IQR* interquartile range.

Cerebellar volume showed a marked age-related reduction across the studied age range. Median cerebellar volume decreased steadily from 136.0 cm³ (IQR 123.6-147.2) in the youngest group to 115.2 cm³ (IQR 107.4-131.1) in subjects older than 85 years. Sex-specific analysis revealed consistently larger cerebellar volumes in males compared to females across all age groups. These differences were statistically significant in nearly all age categories, as assessed by the Mann-Whitney U test. Median infarct volume across the entire cohort was small to moderate, with values ranging between approximately 3.8 and 6.1 cm³ across age groups. No statistically significant differences in stroke volume were observed between the predefined age categories (all *p* > .05). Similarly, comparison between female and male patients revealed no significant sex-related differences in infarct volume within any age group (all *p* > .05). Overall, median infarct volumes demonstrated comparable distributions across age and sex strata, indicating that infarct size was not a confounding factor in the analysis of age- and sex-related infratentorial volumetry. (Table 1).

Brainstem volume exhibited a more moderate age-related decline compared to cerebellar volume. Median brainstem volume increased slightly from early adulthood to middle age, followed by a gradual decrease in older age groups. Median values ranged from 20.1 cm³ (IQR 19.0-23.7) in the youngest group to 20.4 cm³ (IQR 18.2–23.0) in subjects older than 85 years. Males demonstrated significantly larger brainstem volumes than females in most age groups, particularly in middle-aged and older subjects (Table 1). Age-related differences across groups were statistically significant based on one-way ANOVA.

In contrast to neural tissue volumes, the PCF-CSFv, representing cerebrospinal fluid-containing spaces, showed a progressive increase with age. Median PCF-CSFv increased from 28.9 cm³ (IQR 22.7–41.2) in the youngest group to 38.4 cm³ (IQR 22.6–48.0) in the oldest group. Sex-related differences in PCF-CSFv were less pronounced and did not reach statistical significance in most age groups (Table 1), indicating a similar age-related expansion of PCF-CSFv in both males and females.

**Fig. 3:**
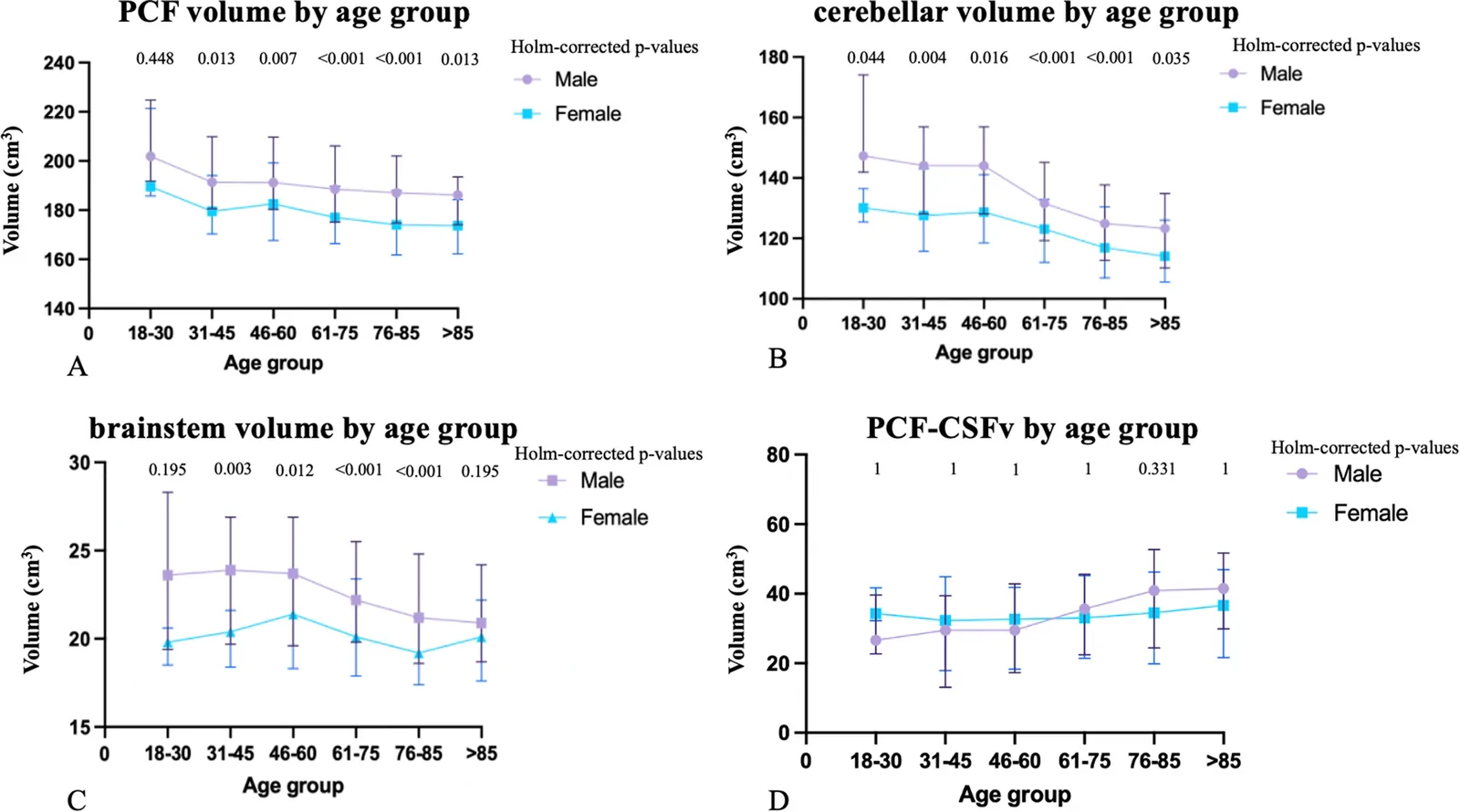
Age-related changes in infratentorial volumetric parameters stratified by sex. Line plots depict median values of (**A**) posterior cranial fossa (PCF) volume, (**B**) cerebellar volume, (**C**) brainstem volume, and (**D**) PCF fluid volume (PCF-CSFv) across predefined age groups. Error bars indicate the interquartile range (25th-75th percentile). Separate curves are shown for male and female subjects. P-values were adjusted separately for each volumetric parameter across the six age groups using the Holm–Bonferroni procedure. Corrected p-values are shown above the corresponding comparisons. A p-corrected value < 0.05 was considered statistically significant

Overall, males exhibited larger PCF, cerebellar, and brainstem volumes compared to females across nearly all age groups (Fig. 3). These differences were most pronounced for cerebellar and brainstem volumes. In contrast, PCF-CSFv did not show consistent sex-related differences, suggesting that age-related enlargement of infratentorial CSF spaces occurs similarly in both sexes.

When analyzing the entire study cohort, males demonstrated significantly larger PCF, cerebellar, and brainstem volumes than females, with all three differences remaining statistically significant after Holm–Bonferroni correction (all p-corrected < 0.001; Table 2). In contrast, PCF-CSFv did not differ significantly between female and male subjects in the overall cohort (p-corrected = 0.438).

**Table 2:** Sex-stratified volumetric characteristics of the overall study cohort

	Female	Male	*p*-corrected
	489 (38.2%)	791 (61.8%)	
PCF volume, median, IQR	177.2 (166.9–190.2)	190 (177.2–207)	< 0.001
Cerebellar volume, median, IQR	121.5 (110.8–132.9)	132 (119.3–145.4)	< 0.001
Brainstem volume, median, IQR	19.9 (17.9–22.4)	22 (19.6–25.5)	< 0.001
PCF-CSFv, median, IQR	33.6 (20.1–45.7)	35.5 (21.2–47.1)	0.438

Corrected p-values represent comparisons between female and male subjects in the overall study cohort using two-sided Mann–Whitney U tests. P-values were adjusted across the four volumetric parameters using the Holm–Bonferroni procedure. Analyses were based on available cases

Abbreviations: *PCF-CSFv* posterior fossa cerebrospinal fluid volume, *PCF* posterior cranial fossa, *IQR* interquartile range

## Discussion

In recent years, clinical neuroscience has steadily transitioned from standardized, population-based reference values toward a more individualized and patient-specific approach. This paradigm shift is particularly relevant for neuroimaging, where quantitative volumetric assessment increasingly informs diagnostic, prognostic, and therapeutic decision-making [8,15,16]. Within this context, the PCF represents a uniquely constrained anatomical compartment in which even subtle volumetric imbalances may have disproportionate clinical consequences [17, 18]. Importantly, infarct volumes were comparable across age groups and between sexes, with no statistically significant differences observed. This finding suggests that lesion size did not act as a confounding factor in the assessment of age- and sex-related differences in posterior cranial fossa volumetry.

The present study, comprising a large cohort of 1,280 adult subjects of different ages and sex, provides comprehensive normative data on age- and sex-related volumetric changes of the PCF and its contents. We observed a marked age-related reduction in cerebellar volume across the studied age range (*p* < .001), accompanied by a smaller but statistically significant decrease in total PCF volume. These findings indicate that aging is associated with a progressive reduction of infratentorial neural tissue, most prominently affecting the cerebellum. Age-related cerebellar atrophy has been extensively investigated in healthy populations [19]. In contrast to previous studies focusing primarily on parenchymal volume loss, our analysis highlights the resulting changes in posterior fossa reserve capacity. From a clinical perspective, the available cerebrospinal fluid space within the posterior cranial fossa may be as important as neural tissue volume itself when evaluating tolerance to acute space-occupying lesions.

Although total PCF volume demonstrated a small but statistically significant decline with increasing age, this reduction was markedly less pronounced than the age-related decrease in cerebellar volume. As a result, PCF-CSFv – the “reserve” spaces - showed a progressive increase across the adult lifespan. From a clinical perspective, even relatively small absolute differences in infratentorial reserve volume may be highly relevant. An additional reserve of approximately ~ 9.5 cm³, as observed between the youngest and oldest age groups, may critically influence the tolerance to space-occupying lesions within the confined PCF.

Cranial growth is completed in early adulthood, subtle age-related changes of the cranial base have been described, such changes may result in a modest reduction of the intracranial compartment without alterations of the external skull dimensions [20, 21].

Importantly, persistent sex-related volumetric differences were observed throughout adulthood. Males exhibited larger PCF, cerebellar, and brainstem volumes compared to females, both within individual age groups and when analyzing the cohort as a whole. This stable sexual dimorphism highlights the limitations of uniform reference values and underscores the necessity of sex-specific normative frameworks, particularly when evaluating infratentorial pathologies.

From a clinical perspective, volumetric relationships within the PCF are of critical importance. The classical Monro-Kellie doctrine conceptualizes the intracranial compartment as a closed system maintaining equilibrium among brain tissue, cerebrospinal fluid, and blood volume [22]. However, this global concept may insufficiently reflect regional infratentorial dynamics. For example, Kuramatsu et al. demonstrated that in patients with spontaneous cerebellar hemorrhage exceeding 15 cm³—within a cohort with a median age of 68.8 years—surgical intervention was associated with improved clinical outcomes [23]. In a similar context, Won et al. demonstrated that in patients with acute cerebellar infarction, an infarct volume of 35 cm³ represented a critical threshold associated with neurological deterioration and the need for surgical intervention, in a cohort with a median age of 68 years [9]. These findings further support the concept that absolute lesion volume plays an important role in clinical decision-making for infratentorial pathology. When interpreted in light of our results, however, such volume-based thresholds should be viewed within the broader framework of individual PCF anatomy.

Our data indicate substantial age-dependent differences in PCF volume and the “reserve” space, suggesting that individual tolerance to space-occupying lesions may vary considerably. Consequently, patients aged 60 and 80 years may differ markedly in their infratentorial volumetric reserve and compensatory capacity, even when lesion volumes are identical. This observation challenges the notion of universally applicable “critical volumes” and highlights the need for a more individualized approach. Our findings suggest that age-related expansion of infratentorial cerebrospinal fluid spaces may provide partial compensation for neural tissue loss; however, this compensatory reserve appears to be limited and highly individual.

The use of neuroimaging volumetric analysis in this study reflects the increasing feasibility of incorporating quantitative measurements into routine clinical practice [16]. Advances in automated and semi-automated segmentation software now enable reliable, reproducible, and near–real-time assessment of posterior cranial fossa structures, thereby facilitating the translation of volumetric research findings into clinically applicable metrics [24]. In the era of personalized medicine, such quantitative approaches are likely to play a central role in tailoring diagnostic thresholds and therapeutic strategies to individual patients.

Future studies using harmonized imaging protocols should compare alternative volumetric approaches, including segmentation-, atlas-, and voxel-based techniques. Such work may enable the derivation of group-averaged volumetric measures and the generation of representative images illustrating structural changes across the cohort.

In conclusion, this study demonstrates that aging and sex significantly influence the volumetric anatomy of the PCF and its contents. The observed imbalance between declining neural tissue volumes and expanding cerebrospinal fluid spaces highlights the importance of individualized infratentorial volumetric assessment. As neuroimaging continues to move toward personalized medicine, quantitative evaluation of PCF anatomy may become an essential component in the assessment and management of infratentorial pathology.

### Limitations

Several limitations should be acknowledged. First, the retrospective and cross-sectional design may have introduced selection bias and does not permit assessment of individual longitudinal changes. Second, the multicenter cohort included MRI and CT examinations acquired using different scanners and clinical protocols, which may have introduced measurement variability despite the use of standardized segmentation methods. Third, the study population consisted of patients with cerebellar stroke and did not include a healthy control group; therefore, the findings should be interpreted as reference data for a vascular population and may not be directly generalizable to healthy individuals. Intracranial volume was not consistently available, precluding normalization for individual head size. In addition, measurements were performed in native anatomical space without MNI-based normalization, and the heterogeneous multimodal dataset did not allow standardized voxel-based morphometry. Finally, intracranial pressure measurements and detailed clinical outcome data were unavailable, preventing direct assessment of the physiological and prognostic relevance of the observed volumetric differences.

## Conclusion

Aging and sex significantly influence the volumetric anatomy of the PCF and its contents. Aging is associated with a reduction of infratentorial neural tissue volumes, particularly the cerebellum, accompanied by a relative expansion of PCF-CSFv. Persistent sex-related differences highlight the need for individualized, age- and sex-specific reference values, supporting the growing role of infratentorial volumetry in personalized neuroimaging assessment.

## Data Availability

No datasets were generated or analysed during the current study.

## Declarations

### Clinical trial registration

Not applicable, as this was a retrospective observational imaging study.
